# Molecular Mechanism of Oxidation of P700 and Suppression of ROS Production in Photosystem I in Response to Electron-Sink Limitations in C3 Plants

**DOI:** 10.3390/antiox9030230

**Published:** 2020-03-11

**Authors:** Chikahiro Miyake

**Affiliations:** 1 Department of Applied Biological Science, Faculty of Agriculture, Graduate School for Agricultural Science, Kobe University, 1-1, Rokkodai, Nada, Kobe 657-8501, Japan; cmiyake@hawk.kobe-u.ac.jp; Tel.: +8-178-803-5851; 2 Core Research for Environmental Science and Technology (CREST), Japan Science and Technology Agency, 7 Goban, Chiyoda, Tokyo 102-0076, Japan

**Keywords:** P700, P700 oxidation system, photorespiration, photosynthesis, Photosystem I (PSI), reactive oxygen species (ROS), reduction-induced suppression of electron flow (RISE), repetitive short-pulse (SP) illumination (rSP illumination treatment)

## Abstract

Photosynthesis fixes CO_2_ and converts it to sugar, using chemical-energy compounds of both NADPH and ATP, which are produced in the photosynthetic electron transport system. The photosynthetic electron transport system absorbs photon energy to drive electron flow from Photosystem II (PSII) to Photosystem I (PSI). That is, both PSII and PSI are full of electrons. O_2_ is easily reduced to a superoxide radical (O_2_**^−^**) at the reducing side, i.e., the acceptor side, of PSI, which is the main production site of reactive oxygen species (ROS) in photosynthetic organisms. ROS-dependent inactivation of PSI in vivo has been reported, where the electrons are accumulated at the acceptor side of PSI by artificial treatments: exposure to low temperature and repetitive short-pulse (rSP) illumination treatment, and the accumulated electrons flow to O_2_, producing ROS. Recently, my group found that the redox state of the reaction center of chlorophyll P700 in PSI regulates the production of ROS: P700 oxidation suppresses the production of O_2_**^−^** and prevents PSI inactivation. This is why P700 in PSI is oxidized upon the exposure of photosynthesis organisms to higher light intensity and/or low CO_2_ conditions, where photosynthesis efficiency decreases. In this study, I introduce a new molecular mechanism for the oxidation of P700 in PSI and suppression of ROS production from the robust relationship between the light and dark reactions of photosynthesis. The accumulated protons in the lumenal space of the thylakoid membrane and the accumulated electrons in the plastoquinone (PQ) pool drive the rate-determining step of the P700 photo-oxidation reduction cycle in PSI from the photo-excited P700 oxidation to the reduction of the oxidized P700, thereby enhancing P700 oxidation.

## 1. Introduction

Numerous researchers have shown that, in photosynthetic organisms, oxidative damage due to enhanced production of reactive oxygen species (ROS) occurs when environmental stress (e.g., extreme low/high temperatures, high salinity, and oligotrophic inorganic components) decreases the photosynthetic efficiency [1,2,3,4,5,6,7,8,9,10,11,12,13,14,15,16,17,18,19,20,21]. Much of this research has focused on the Mehler reaction, whereby a superoxide radical (O_2_**^−^**), one of the most important ROS species, is formed via a one-electron reduction of O_2_ in Photosystem I (PSI) of the chloroplast thylakoid membranes. In addition to PSI, ROS production in Photosystem II (PSII) has long been studied, and numerous studies have been published regarding its relationships to oxidative damage in PSII across oxygenic photosynthetic organisms [22,23,24,25,26,27,28,29]. This review focuses on our recent findings regarding the molecular mechanisms of the production and suppression of ROS in PSI.

We try to answer the following questions in this review: “Is the excess accumulation of electrons in PSI truly harmful to photosynthetic organisms?” and “Does ROS production occur in vivo?” Driever and Baker [30,31] and Ruuska et al. [30,31] reported that the in vivo activity of the Mehler reaction is too small compared to the electron flux in photosynthesis. Additionally, considering the photosynthetic capacity of many photosynthetic organisms, the photosynthetic rate is saturated at a light intensity level corresponding to approximately 25% of natural sunlight or lower [2,3,31,32,33,34,35,36,37]. This indicates that photosynthesis regularly proceeds under conditions of an excess photon supply, which can cause the photosynthetic electron transport system to be full of electrons. To address this issue, we asked “How do photosynthetic organisms manage to escape from the accumulation of electrons in PSI?” We then developed a method for suppressing photosynthesis and imitating the accumulation of electrons in PSI. Using this approach, we succeeded in showing how ROS are produced and suppressed in PSI [38]. In what follows, the robust and universal characteristics of the suppression of ROS production in photosynthetic organisms are discussed.

## 2. The Accumulation of Electrons in the PSI Acceptor Side Promotes ROS Production and Oxidative Damage

**Figure 1:**
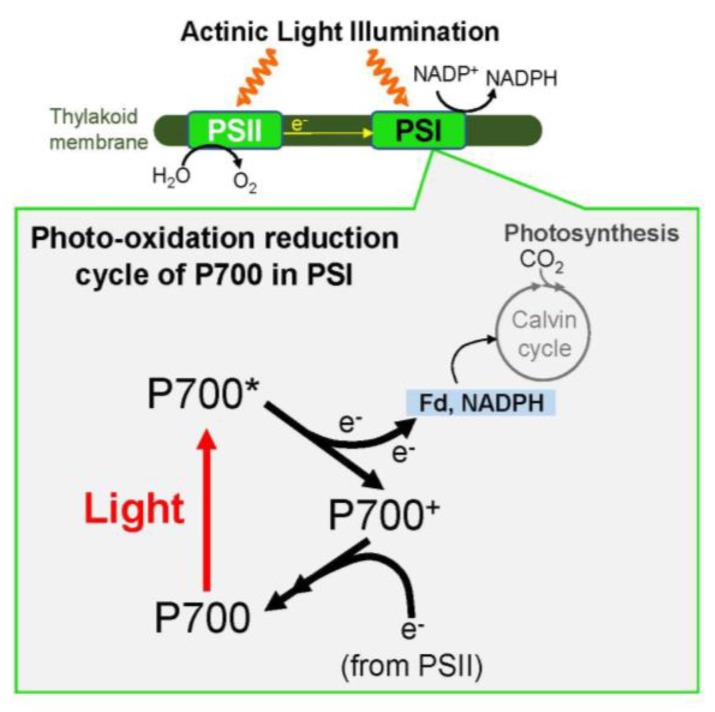
The photo-oxidation reduction cycle of P700 in Photosystem I (PSI). The reaction center chlorophyll P700 in PSI absorbs the light energy, and the P700 is excited to P700*. The P700* donates electrons to the electron carrier, A_0_, and concomitantly produces the oxidized P700, P700^+^. The P700^+^ is reduced by electrons from Photosystem II (PSII) through plastoquinone (PQ), the cytochrome (Cyt) *b6/f*-complex, and plastocyanin (PC). The reduced PC directly donates electrons to P700. There are then P700 turnovers in the photo-oxidation reduction cycle of P700 in PSI. The electron on A_0_ flows to NADP^+^ to produce NADPH through the electron carriers A_1_, F_x_, F_A_/F_B_, and Fd. In the photosynthetic linear electron flow, electrons extracted from H_2_O oxidation in PSII flow to NADP^+^ for the production of NADPH.

**Figure 2:**
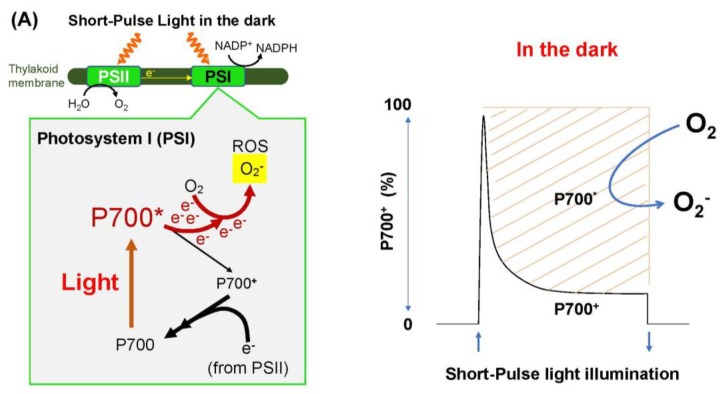

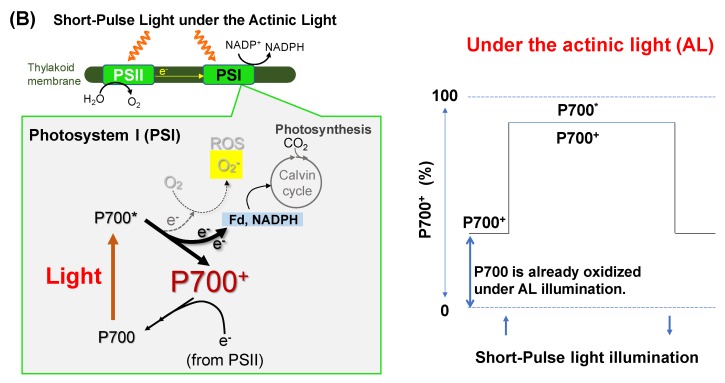
The kinetics of oxidized P700 during short-pulse (SP) illumination in the dark and under actinic light. (**A**) An SP illumination (20,000 μmol photons m^−2^ s^−1^, 400 ms) to intact leaves in the dark rapidly oxidizes the reaction center chlorophyll P700 in PSI from the fully reduced state to the maximally oxidized state. Afterwards, oxidized P700 (P700^+^) is reduced by electrons from PSII, with the accumulation of the excited state of P700 (P700*), as shown by the increase in shaded area. P700* can donate an electron to O_2_, thereby producing a superoxide radical (O_2_**^−^**) through the one-electron reduction by the electron carriers, A_0_/A_1_, F_x_, and F_A_/F_B_, localized at the acceptor side of PSI. (**B**) In actinic light (AL) illumination, before SP illumination, P700 is already oxidized to P700^+^. SP illumination further oxidizes P700 to its maximum, which is lower than the oxidation obtained in the dark, because P700* accumulates under the steady-state AL illumination. P700^+^ produced by SP illumination is not reduced, because P700 in PSI experiences turnover with the rate-determining step of P700^+^ reduction in the P700 photo-oxidation reduction cycle. The Y-axis shows the oxidation ratio of P700 from 0% to 100%, and the X-axis shows the time for SP illumination: upward arrow: SP illumination starts; downward arrow: SP illumination stops.

**Figure 3:**
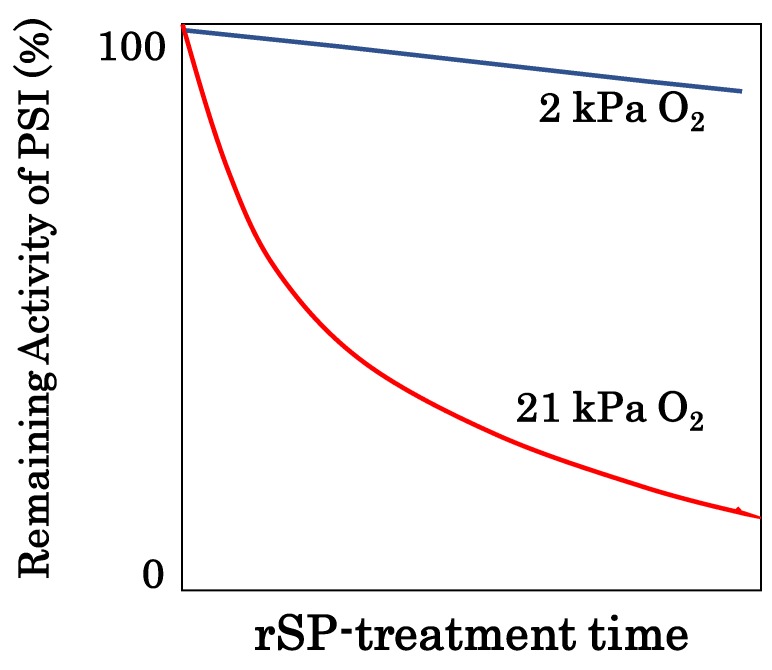
Repetitive short-pulse (rSP) illumination treatment inactivates PSI. In angiosperms, rSP illumination treatment of the intact leaves inactivates PSI activity [38]. The inactivation is suppressed under low O_2_ conditions (2 kPa O_2_). Short-pulse illumination reduces O_2_ to a superoxide radical, O_2_**^−^**, and the accumulated O_2_**^−^** from rSP illumination treatment oxidatively attacks PSI components. Red line: the PSI activity gradually decreases as rSP illumination treatment proceeds under atmospheric O_2_ (21 kPa) condition; blue line: PSI inactivation by rSP illumination treatment is suppressed under low O_2_ (2 kPa) condition.

In the photosynthetic electron transport system, the reaction center chlorophyll P700 in PSI functions in the photo-oxidation reduction cycle (Figure 1). Ground state P700 is photoexcited to produce the excited P700 (P700*), which then donates an electron to the primary electron carrier (A_0_) to produce the oxidized P700 (P700^+^). Subsequently, P700^+^ accepts an electron from the reduced plastocyanin (PC) to regenerate P700. The oxidized PC in turn is reduced by the electrons coming from PSII. The electron in the reduced A_0_ flows to ferredoxin (Fd) through the electron carriers: A_1_, F_x_, and F_A_/F_B_ [39]. If a light pulse (light intensity: 20,000 μmol photons m^−2^ s^−1^; pulse duration: 400 ms) is applied to C3-sunflower intact leaves in the dark under atmospheric conditions, all P700 molecules are oxidized to P700^+^ (Figure 2A). P700^+^ then rapidly decreases during the light pulse, which is due to the occupation of P700* in the photo-oxidation reduction cycle of P700, indicating the accumulation of electrons at the acceptor side of PSI. If the light pulse is applied every 10 s on C3-sunflower intact leaves (i.e., repetitive short-pulse (rSP) illumination treatment), PSI is deactivated (Figure 3) [38]. Conversely, PSII is not deactivated at all. PSI deactivation is all suppressed when atmospheric O_2_ partial pressure drops to 2 kPa. The atmospheric rSP illumination treatment then produces a superoxide radical (O_2_**^−^**) in PSI [32,40,41,42,43]. These results suggest that the electrons accumulated on the PSI acceptor side during one light pulse are used for O_2_ reduction, which triggers the O_2_**^−^** production reflected as P700* accumulation. O_2_**^−^** accumulation occurring over time with rSP illumination treatment indicates progressive PSI oxidative damage to the photosynthetic organism. PSI inactivation due to rSP illumination treatment represents a case of photosynthetically induced CO_2_ fixation inactivation [9,35,38,44]. Unlike PSII, PSI requires approximately one week for functional recovery following inactivation [7,9,35,36,45,46]. Therefore, PSI inactivation can be fatal to plant growth [7,35,36,42]. Exposure of plants to low temperatures also inactivates PSI and the recovery of PSI function can take several weeks.

## 3. ROS Production Is Suppressed by Oxidation of P700

As described above, ROS-dependent oxidative damage in PSI was shown in vivo. Next, a new question arose: essentially, how do plants survive under natural sunlight? The answer was quickly obtained. PSI inactivation by rSP illumination treatment was suppressed under illumination by actinic light (AL) [38]. Furthermore, the extent of PSI inactivation decreased with increasing AL intensity. In other words, the light-induced inactivation of PSI was prevented by light itself. The effect of AL illumination was that P700 was oxidized to P700^+^ (Figure 2B). Increasing AL intensity increased P700^+^ in the P700 photo-oxidation reduction cycle. Afterwards, P700^+^ accumulation prevented P700* accumulation during SP illumination (Figure 2B). In other words, high levels of P700^+^ were maintained, even during light pulses. Thus, the role of AL illumination from the perspective of the P700 photo-oxidation reduction cycle was made clear: both P700* accumulation in the dark and P700^+^ accumulation under AL illumination during an SP illumination, indicate the rate-determining step (RdS) transition in the P700 photo-oxidation reduction cycle. In the dark, P700* oxidation is rate-limited, whereas under AL illumination, P700^+^ reduction is rate-limited. AL illumination suppresses rSP-illumination-treatment-induced PSI inactivation through the rate-determining step transition mechanism. That is, AL illumination suppresses the accumulation of P700*, which can donate electrons to O_2_ to produce O_2_**^−^** in PSI. Subsequently, we found the robust effect of AL illumination, which was that P700 was oxidized and suppressed PSI inactivation by rSP illumination treatment [42].

Numerous studies have reported the oxidation of P700 in PSI under high intensity AL or low CO_2_ conditions [33,34,47,48,49,50,51,52,53,54]. Accumulated P700^+^ during the P700 photo-oxidation reduction cycle absorbs excess photon energy and dissipates it as heat [55,56,57]. Concomitantly, P700^+^ accumulation inhibits O_2_ reduction, producing O_2_**^−^** by suppressing P700* accumulation [38]. This is the molecular mechanism that reduces the rate of the Mehler reaction in intact leaves under AL illumination. If P700 is not subsequently oxidized, ROS production will increase, due to the promotion of the Mehler reaction, and PSI will stop functioning [38,42,58].

## 4. Molecular Mechanism of Oxidation of P700 in Photosystem I under AL Illumination

In photosynthesis, a light reaction functions with a dark reaction in a tightly coupled state. For example, the light reaction proceeds in the thylakoid membranes of chloroplasts in C_3_-plants, where photosynthetic linear electron flow produces NADPH and ATP. The dark reaction proceeds in both photosynthesis and photorespiration, where both NADPH and ATP are used for the regeneration of ribulose 1,5-bisphosphate (RuBP) (one of the substrates for RuBP carboxylase/oxygenase which is consumed in the dark reaction). In photorespiration, reduced Fd is used in chloroplasts. Electron flow, which produces both NADPH and the reduced Fd, and proton flow, which produces ATP in the light reaction, are coupled, with both e^-^ flow and H^+^ flow required for the consumption of NADPH, the reduced Fd, and ATP in the dark reaction. This tight coupling of the light reaction with the dark reaction can cause a dangerous situation in which ROS are produced in the photosynthetic electron transport system. The limitation of the dark reaction is the limitation of the light reaction, which can lead to the accumulation of electrons in the photosynthetic electron transport system. For example, in the photo-oxidation reduction cycle of P700 in PSI, electrons would accumulate at the acceptor side of PSI, where O_2_ can be reduced to O_2_**^−^**, a ROS. As described above, unless P700 in PSI is oxidized, PSI suffers from oxidative damage.

We show below how the light reaction tightly couples with the dark reaction in photosynthesis. We later show how P700 is oxidized in response to the limitation of the dark reaction, for example, in drought, low temperature, and high light situations.

### 4.1. Robust Relationships Support Tight Coupling between the Light and Dark Reactions in Photosynthesis

**Figure 4:**
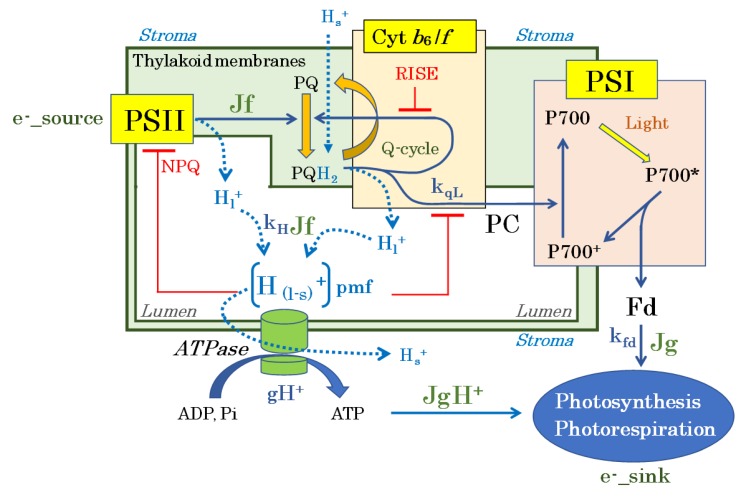
Tight coupling between the light and the dark reactions in photosynthesis of C3 angiosperms. Blue arrows: electron flow; blue dotted arrows: H^+^ flow; Hs^+^: stroma H^+^; H_l_^+^: lumen H^+^; [H(_l − s_)^+^]: difference in H^+^ concentration (ΔpH) between the stroma and the lumen, i.e., the proton motive force (pmf). Photosystem II (PSII): electron source. P700 functions in the photo-oxidation reduction cycle to catalyze the electron flow from plastocyanin (PC) to Fd in Photosystem I (PSI). P700 is photoexcited to P700*, which donates electrons to Fd through electron carriers (A_0_, A_1_, F_A_/F_B_, and F_X_) to produce P700^+^. In turn, P700^+^ is reduced by electrons from PSII through plastoquinone (PQ), the Cyt *b*_6_/*f* complex, and PC. The Q-cycle in the Cyt *b*_6_/*f* complex transfers electrons from reduced PQ along two routes: first, PC; second, oxidized PQ. Enhanced reduction of PQ suppresses the reduction of oxidized PQ in the Cyt *b*_6_/*f* complex, which slows down the activity of the Q-cycle, and the electron flux from the Cyt *b*_6_/*f* complex to P700^+^ in PSI. H^+^ conductance (gH^+^) is an apparent rate constant that depends on the concentrations of ADP, Pi, ATPase, and the catalytic constant (k_(ATPase)_). NPQ, non-photochemical quenching of chlorophyll fluorescence, is activated by the acidification of the lumenal space of the thylakoid membrane, which is reflected by the increase in pmf. NPQ suppresses the quantum yield of PSII, which is reflected by the electron flux in photosynthetic linear electron flow, Jf; the pmf also suppresses the oxidation activity of reduced PQ by the Cyt b_6_/f complex. Tight coupling of the light reactions (the production of electron and proton flows) to the dark reaction (the consumption of electrons and protons in both photosynthesis and photorespiration) yields a robust relationship whereby electron flux (Jf) is equal to the electron flux in electron sinks (photosynthesis and photorespiration) expressed as Jg. The pmf is the driving force to synthesize ATP by ATP synthase using ADP and Pi, and the pmf-dissipation rate (vH^+^) is expressed as gH^+^ × pmf. As a robust model, J_f_ = vFd = J_g_ [61]; vFd is the oxidation rate of reduced Fd; k_Jf_ × Jf = gH^+^ × pmf [64,65]. vH^+^ = gH^+^ x pmf = J_gH+_ [66]. J_g_ and J_gH+_ mutually determine each other. Meanwhile, pH decreases in the lumenal space of the thylakoid membrane during the photo-oxidation of water in PSII and the Q-cycle in the Cyt *b*_6_/*f* complex. The decrease in pH (i.e., increased pmf) acts as the driving force for ATP production by ATPase.

Much evidence has accumulated that clarifies a robust tight coupling between the light and dark reactions in C3 photosynthesis (Figure 4). For instance, Genty et al. [59] reported a positive linear relationship with an origin of zero between the photosynthetic linear electron flow rate (J_f_) in the light reaction and the photosynthetic electron consumption rate (J_g_) based on NADPH consumption—in the dark reaction [59]. Consistently, a positive linear relationship with an origin of zero was also found between the photosynthetic linear electron flow rate (J_f_) in the light reaction and the electron consumption rate (J_g_)—based on NADPH consumption and reduced Fd—in the dark reaction, where photosynthesis and photorespiration both occur (Equation (1)) [30,31,60]. These results indicate that photosynthetic linear electron flow is the sole driver of both photosynthesis and photorespiration. In other words,

Jf = Jg(1)

Recently, a positive linear relationship with an origin of zero between the reduced Fd oxidation rate (vFd) and the photosynthetic linear electron flow rate (J_f_) was found (Equation (2) [61]). These results indicate that electron flow via Fd is driven by both photosynthesis and photorespiration. In other words,

vFd = k_fd_ × Fd^−^ = Jg (2)

The constant k_fd_ is the apparent rate constant of the reduced Fd oxidation rate, while Fd^−^ is the amount of reduced ferredoxin.

A positive linear relationship with an origin of zero was reported between Jf and the pmf production rate ((vH^+^ = gH^+^ × pmf (gH^+^, proton conductance) (Equation (3)) [62,63,64,65]). These results indicate that photosynthetic linear electron flow provides the formation of pmf. In other words,

k_H_^+^ × Jf = vH^+^ (3)

The rate constant k_H+_ reflects lumen H^+^ accumulation efficiency, which depends on H_2_O oxidation in PSII and on the Q-cycle in the Cyt *b*_6_/*f* complex. Furthermore, a positive linear relationship with an origin of zero was found between vH^+^ and the proton usage rate (JgH^+^), due to the regeneration of ATP required for both photosynthesis and photorespiration (Equation (4)) [66]. These results indicate that vH^+^ also reflects the pmf usage rate during photosynthesis and photorespiration. In other words,

vH^+^ = JgH^+^ (4)

Furthermore, a positive linear relationship with an origin of zero was found between JgH^+^ and J_g_ (=J_f_) (Equation (5)) [66]. These results indicate that JgH^+^ was determined by the electron consumption rate (J_g_), based on the consumption of both reduced Fd and NADPH. In other words,

JgH^+^ = k_jg_ × Jg = k_H_^+^ × Jf (5)

Equation (5) indicates that there is a tight coupling between the light and dark reactions during photosynthesis and photorespiration. Constant k_jg_ is the coefficient that links e^-^ flow with H^+^ flow during photosynthesis and photorespiration. These findings indicate that the operation of the light reaction is driven by the dark reaction and vice versa, and provide a concrete explanation for the observed decrease in J_f_ under environmental stress, when the photosynthetic rate decreases due to a dark reaction rate limitation (i.e., when JgH^+^ and J_g_ decrease).

Tight coupling between the light and dark reactions, as shown in Equation (5), can cause the accumulation of electrons at the acceptor side of PSI and the accumulation of protons in the lumenal space of thylakoid membranes under stress conditions, e.g., drought, low temperatures, and high intensity of light, which decrease the photosynthesis efficiency [2,3,8,67,68,69]. For example, under constant light intensity, decreases in both Jg and JgH^+^ decrease Jf. These situations cause a reduction in PQ and the non-photochemical quenching of chlorophyll fluorescence, both of which reflect the accumulations of electrons and protons in the photosynthetic electron transport system [32,34,42,58,70,71,72].

However, the electrons do not accumulate at the acceptor side of PSI; P700 is oxidized under stress conditions [32,34,49,50,53,72]. From the model of the photo-oxidation reduction cycle of P700, the reduction of P700^+^ must be the rate-determining step to accumulate the oxidized P700 in PSI. The suppression of electron fluxes in both light and dark reactions does not limit the reduction of P700^+^ in the cycle. That is, the electron flux to the oxidized P700 should be suppressed such that P700 is oxidized.

### 4.2. How Do Protons and Electrons Accumulate in Both the Lumenal Space of Thylakoid Membranes and the PQ Pool in Response to the Suppression of the Dark Reaction?

Generally, it is proposed that the electron flux from PQ to P700 in PSI is regulated by the oxidation activity of the reduced PQ (PQH_2_) by the cytochrome (Cyt) *b*_6_/*f*-complex [48,67,70,71,73,74,75,76,77,78,79,80,81]. The PQH_2_ oxidation activity by the Cyt *b*_6_/*f*-complex decreases with a lowering pH. Therefore, the accumulation of protons in the lumenal space of thylakoid membranes suppresses the electron flow to the oxidized P700 in PSI, which is known as photosynthesis control [67,73,74]. Furthermore, the overreduction of PQ inhibits the Q-cycle in the Cyt *b*_6_/*f*-complex, and contributes to the oxidation of P700 in PSI, which is known as the reduction-induced suppression of electron flow (RISE) [70,71]. We propose that the RISE is also involved in photosynthesis control. To clarify the molecular mechanism of oxidation of P700 in PSI upon exposure to the limitation of photosynthesis, we must understand how protons and electrons accumulate in both the lumenal space and the PQ pool in the photosynthetic electron transport system in response to the activities of both photosynthesis and photorespiration.

The induction mechanism of proton gradient (ΔpH) formation across the thylakoid membrane, the accumulation of protons in the lumenal space of thylakoid membranes, is shown as below: ΔpH formation is observed as a pmf increase [65,82,83]. In general, ΔpH mainly occupies the pmf at low photosynthetic efficiency, e.g., high light and low CO_2_ conditions. The velocity of change in pmf [d(pmf)/dt] is determined by the difference between the pmf generation rate and the pmf decay rate. The pmf generation rate depends on the photosynthetic linear electron flow rate (Equation (3)), and the pmf decay rate depends on the ATP production rate, which is driven by both photosynthesis and photorespiration (Equation (4)): thus, the velocity of change in pmf is as shown (Equations (6) and (7)).

d(pmf)/dt = k_H_^+^ × Jf − vH^+^ (6)

= k_H_^+^ × Jf − gH^+^ × (pmf) (7)

The rate constant k_H+_ reflects the H^+^ accumulation in the luminal space of thylakoid membranes, which is driven by photosynthetic linear electron flow. At the steady state, d(pmf)/dt = 0, and the pmf production rate is k_H_^+^ × Jf. This depends on H_2_O oxidation in PSII and on Q-cycle rotation in the Cyt *b*_6_/*f* complex. Furthermore, the pmf decay rate, vH^+^, is expressed as gH^+^ × (pmf). The gH^+^, H^+^ conductance, is a rate constant that reflects the apparent rate constant of pmf decay. The vH^+^ also reflects the pmf dissipation rate, and vH^+^ can be replaced with JgH^+^ as follows (Equation (8)):

d(pmf)/dt = k_H_^+^ × Jf − JgH^+^ (8)

We reveal that, in a steady state where d(pmf)/dt = 0, based on Equations (7) and (8), Equation (9) was obtained.

k_H+_ × Jf = vH^+^ (= gH^+^ × pmf) = JgH^+^ (9)

Equation (9) shows that the photosynthetic linear electron flow activity in the light reaction links photosynthesis and photorespiration activity in the dark reaction through pmf formation and dissipation. The dependence of pmf on Jf, gH^+^, and JgH is shown as follows (Equations (10) and (11)).

pmf = (k_H_^+^ × Jf)/gH^+^ (10)

= JgH^+^/gH^+^ (11)

Based on this model, we will discuss the molecular mechanism of proton accumulation in the lumenal space of thylakoid membranes in response to the activities of photosynthesis and photorespiration. For example, if the extent of the decrease in the dark reaction rate, shown as the decrease in both Jg and JgH^+^, was smaller than that in gH^+^, then pmf increased. This is how protons accumulate in the lumenal space of thylakoid membranes.

We consider the accumulation of electrons in the photosynthetic electron transport system, reflected as the redox states of both PQ and Fd. The reduced state of PQ is evaluated by a parameter of chlorophyll fluorescence, 1 − qL [72,84]. The qL shows the oxidation state of PQ in thylakoid membranes. A higher value of 1 − qL shows the high accumulation of electrons in the PQ pool. The oxidation rate of the reduced PQ can be expressed as kqL × (1 – qL). Additionally, the oxidation rate of the reduced Fd (Fd^−^) is expressed as k_Fd_ × Fd^−^. Both kqL and kFd are the apparent rate constants. In the light reaction, the electron fluxes for the oxidations of both the reduced PQ and the reduced Fd are equal to Jf, as shown in the equation (12).

Jf = kqL × (1 – qL) = k_Fd_ × Fd^−^ = Jg (12)

The kqL is given a positive value and is expected to decrease by two factors: first, kqL decreases with a decrease in pH in the lumenal space of thylakoid membranes (photosynthesis control) [67,85,86]; second, kqL decreases by the RISE [70,71]. Generally, a decrease in Jg increases the value of 1 – qL [34,72]. These facts indicate, in response to the decrease in electron sink activity (both photosynthesis and photorespiration), that the extent of the decrease in kqL is larger than that in Jg. That is, from Equation (12), we can clearly understand why 1 – qL increases. This is how electrons accumulate in the PQ pool of thylakoid membranes.

On the other hand, a decrease in Jg does not induce the reduction of Fd [61]. For example, lowering the intercellular partial pressure of CO_2_ decreases the photosynthesis rate, and Jg decreases; however, the reduced level of Fd either does not change or decrease. These facts show that the extent of the decrease in k_Fd_ was almost the same as that in Jg. The k_Fd_ depends on both the activity of Fd^−^NADP^+^ oxidoreductase (FNR) and NADP^+^ regeneration efficiency. The decrease in the photosynthesis rate lowers the NADP^+^ regeneration rate, which decreases k_Fd_.

### 4.3. How Do the Accumulated Protons in the Lumenal Space and Electrons in the PQ Pool Oxidize P700 in PSI?

**Figure 5:**
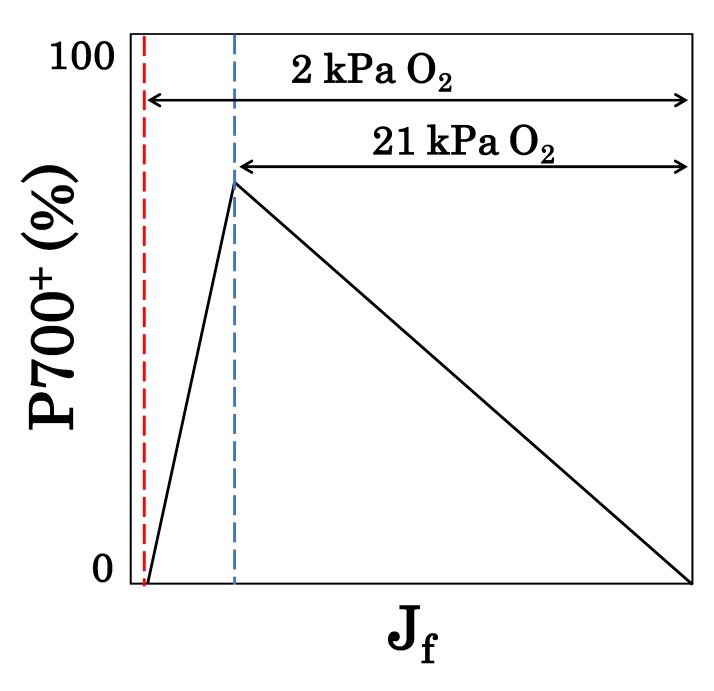
Dependence of oxidized P700 (P700^+^) induction on electron flux in photosynthetic linear electron flow in intact leaves. Generally, P700 is oxidized with a decreasing electron flux of photosynthetic linear electron flow (J_f_) by lowering CO_2_ partial pressure. At 21 kPa O_2_, when both photorespiration and photosynthetic CO_2_ fixation are functioning, P700^+^ shows the maximum value at the CO_2_ compensation point, while J_f_ shows the minimum value (blue dotted line). The horizontal arrow at 21 kPa O_2_ shows the range over which J_f_ is driven by both photosynthetic CO_2_ fixation and photorespiration. At 2 kPa O_2_, when no detectable photorespiration occurs and photosynthetic CO_2_ fixation functions normally, P700^+^ starts to decrease to the minimum at the CO_2_ compensation point after reaching its maximum value, while J_f_ reaches its minimum value (red dotted line). The horizontal arrow at 2 kPa O_2_ shows the range over which J_f_ is driven by photosynthetic CO_2_ fixation.

The decrease in Jf (=Jg) shows the negative linear relationship with P700^+^ (Figure 5) [42,87]. The decrease in photosynthesis efficiency induces the oxidation of P700 in PSI. As described above, the suppression of photosynthesis enhances both the pmf and the RISE, which would cause the reduction of P700^+^ in the photo-oxidation reduction cycle of P700 in PSI to be the rate-determining step. The decrease in Jg lowers Jf in the tight coupling of the light reaction with the dark reaction. Here, we have to pay an attention to the magnitude of the decrease in Jf. The extent of the decreases in gH^+^ and kqL are larger than that in Jg, so the pmf and (1 – qL) increase. We also must consider that the magnitude of Jf would be downregulated by both the pmf and the RISE, such that the reduction of P700^+^ should be the rate-determining step in the photo-oxidation reduction cycle of P700 in PSI. Jf must be lower than the theoretical value of Jg obtained by the suppression of photosynthesis. For example, if Jf decreases to the same extent as the decrease in Jg in the response to the decrease in Ci, the photo-excited P700 (P700*) would accumulate, because the efficiency of photo-excitation of P700 to P700* does not change at a constant intensity of AL. This deduction contradicts the experimental facts; that is, that suppressed photosynthesis induces the oxidation of P700.

The RISE was evidenced to have the potential to stop the electron flow in the light reaction and to oxidize P700 in PSI of cyanobacteria [70,71]. Flavodiiron protein (FLV) catalyzes the reduction of O_2_ to H_2_O using NADPH in the cyanobacteria [88,89]. The electron flux in the FLV reaction is so high that it alternates the electron flux in photosynthesis under CO_2_-deficient conditions [88,90]. The cyanobacteria deficient in FLV show suppressed photosynthesis, with the accumulation of electrons in the PQ pool [70]. Furthermore, illumination by SP light reduces PQ almost transiently with the suppression of O_2_ evolution under steady-state conditions [70]. FLV can oxidize PQ even though photosynthesis can function at a higher activity. FLV keeps PQ in an oxidized state so as not to induce the RISE for the full activity of photosynthesis, if enough CO_2_ is supplied to cyanobacteria.

Furthermore, we found that inductions of the pmf and RISE oxidize P700 in PSI of the C3-plant wheat leaves [42,87]. A decrease in the intercellular partial pressure of CO_2_ lowers the photosynthetic rate and increases the photorespiration rate. However, the photorespiration rate cannot reach the value theoretically derived from Rubisco kinetics at lower CO_2_ [37,91]. The induction of the pmf and RISE lowers the activity of the light reaction, which causes the activity of the dark reaction, the photorespiration rate, to be suppressed, even though the potential activity of the dark reaction exceeds the suppressed activity of the light reaction. These results show that the oxidation of P700 requires the suppression of electron flux in the light reaction much more so than in the dark reaction; thus, the suppressed activity in the dark reaction is lower than the theoretical potential activity. As a result, the reduction of P700^+^ becomes the rate-determining step in the P700 photo-oxidation reduction cycle. This is the essence of the molecular mechanism of P700 oxidation.

On the other hand, the induction of the pmf and RISE does not always oxidize P700 in response to the limitation of photosynthesis. Unless the electron flux of the light reaction dips below the potential electron flux of the dark reaction, which is, theoretically, expected from the limited activity of photosynthesis, the limitation of the PSI acceptor side is so strong that P700 is not oxidized (Figure 5). When photosynthesis is severely limited, where both photosynthesis and photorespiration functions are suppressed, P700 cannot be oxidized because the oxidation of P700* becomes the rate-determining step in the P700 photo-oxidation reduction cycle.

### 4.4. Suppression of the Production of ROS in PSI by the P700 Oxidation System

**Figure 6:**
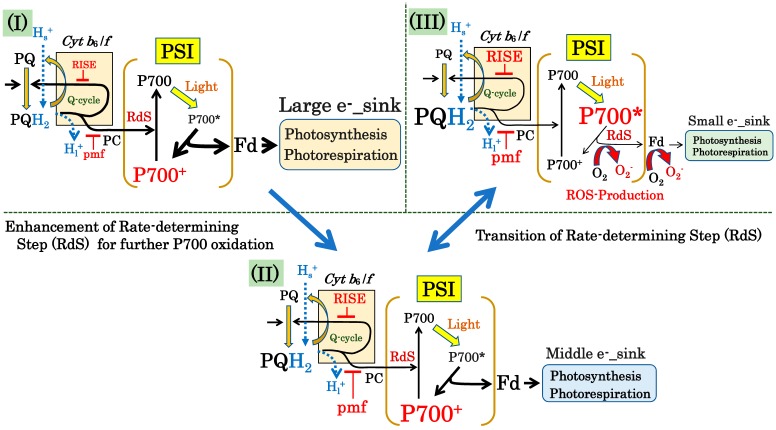
The P700 oxidation system and ROS production mechanism. The photosynthetic linear electron flow in the light reaction is tightly coupled to the dark reaction, photosynthesis and photorespiration (electron sinks). The P700 oxidation system enhances the rate-determining step of P700^+^ reduction to stimulate the oxidation of P700 in PSI, in response to the decrease in electron sinks. Once exposed to a further decrease in electron sinks, P700^+^ reduction cannot remain the rate-determining step, and the rate-determining step transitions from P700^+^ reduction to the oxidation of P700*. Three distinct scenarios of the rate-determining step can take place under saturated light intensity: In Case (I) with the large electron sink, both the pmf and RISE are induced by the two electron sinks: photosynthesis and photorespiration. Afterwards, the oxidation activity of the reduced PQ by the Cyt *b*_6_/*f*-complex is suppressed to decrease the reduction rate of P700^+^. As a result, the photo-oxidation reduction cycle of P700 in PSI turns over with the rate-determining step of P700^+^ reduction. In Case (II) with the medium-sized electron sink, PQ is further reduced, and the pmf is further accumulated, compared to Case (I), by the decreased gH^+^ and k_qL_. The electron flux from the reduced PQ to the oxidized P700 is then further suppressed. The oxidation of P700 becomes the rate-determining step in the photo-oxidation reduction cycle of P700 in PSI. In Case (III) with the small electron sink, the further induced pmf and RISE cannot decrease the reduction rate more than the suppression of the oxidation of P700* due to the smaller electron sink, compared to Case (II). Afterwards, P700* accumulates. As a result, the photo-oxidation reduction cycle of P700 in PSI turns over with the rate-determining step of P700* oxidation. From Case (II) to Case (III), the rate-determining step transitions from the reduction of P700^+^ to the oxidation of P700* in the photo-oxidation reduction cycle of P700 in PSI. The accumulated P700* increases the chance of the reduction of O_2_ to O_2_**^−^**, the production of ROS at the acceptor side of PSI. The reduced electron carriers in PSI—A_0_, A_1_, F_x_, F_A_/F_B_, and the reduced Fd—can react with O_2_ to produce O_2_**^−^** [2,39,40,41,92,93].

We summarize the molecular mechanism of P700 oxidation in PSI of chloroplasts (Figure 6). Both PSI and PSII absorb photon energy to excite the reaction center chlorophylls P680 (PSII) and P700 (PSI). Similarly to P700 in PSI, P680 undergoes a photo-oxidation reduction cycle. The ground state of P680 is photo-excited to P680*. The P680* donates electrons to PQ through electron carriers in PSII. Additionally, then, the electrons start to flow from the reduced PQ to PSI, through the Cyt *b*_6_/*f*-complex, and plastocyanin (PC). During the electron flow, H^+^ from both H_2_O oxidation in PSII and the reduced PQ (PQH_2_, plastoquinol) oxidation of the Cyt *b*_6_/*f*-complex accumulate in the lumenal space of thylakoid membranes. The accumulated protons are the pmf to drive ATP synthase to produce ATP in the stroma. On the other hand, NADPH is produced by ferredoxin-NADP oxidoreductase (FNR) at the acceptor side of PSI. ATP, NADPH, and the reduced Fd are used for the regeneration of RuBP in both photosynthesis and photorespiration. The regeneration rates of NADP^+^, the oxidized Fd, and ADP are determined by the activities of both photosynthesis and photorespiration.

As shown in Equations (5), (9), and (12), the light reaction tightly couples with the dark reaction. The suppression of photosynthesis decreases Jg or JgH^+^. Afterwards, the regeneration rates of ADP, Pi, and NADP^+^ decrease. That is, gH^+^ decreases to increase the pmf, and kqL decreases to increase (1 – qL), which induces the RISE. Both the pmf and the RISE lower kqL, as reflected in the suppressed activity of the reduced PQ oxidation by the Cyt *b*_6_/*f*-complex. NPQ induced by the pmf also downregulates the quantum yield of PSII. The suppressed electron flow from PSII to the oxidized P700 also causes the reduction of P700^+^ in the photo-oxidation reduction cycle to be the rate-determining step, which leads to the oxidation of P700.

We compared the rate-determining step in the photo-oxidation reduction cycle of P700 in PSI in response to the limitation of electron sink activities and the photosynthesis and photorespiration activities at high light conditions (Figure 6). In Case (I), we set the electron sink to a large size. The black arrows show the electron flow in the photosynthetic electron transport system from the redox reaction of PQ to the electron sink, including both photosynthesis and photorespiration, through the Cyt *b*_6_/*f*-complex, PC, and P700 in PSI and Fd. The width of the black arrows reflects the magnitude of the apparent rate constant in each elementary reaction. The rate-determining step of the P700 photo-oxidation reduction cycle in PSI is the reduction of P700^+^, leading toward to the oxidation of P700^+^. Both the pmf and RISE contribute to the suppression of the Q-cycle activity of the Cyt *b*_6_/*f*-complex, which causes the reduction of P700^+^ to be the rate-determining step.

In Case (II), the electron sink is set to a medium size. Environmental stress, e.g., drought and/or low temperatures, can suppress photosynthetic activities. That is, both the pmf and RISE increase in response to the decrease in both gH^+^ and kqL, as determined by Equations (11) and (12). The apparent rate constant for the reduction of P700^+^ further decreases, compared to Case (I). The magnitude of the apparent rate constant for P700^+^ reduction is lower than the apparent constant for P700* oxidation. The increase in the suppression of the Q-cycle in the Cyt *b*_6_/*f*-complex enhances the rate-determining step, the reduction of P700^+^, in the P700 photo-oxidation reduction cycle of PSI.

In Case (III), the electron sink is smaller than the medium size in Case (II). The activities of both photosynthesis and photorespiration are further suppressed, compared to Case (II). However, the rate-determining step leads to the elementary reaction of the oxidation of the photo-excited P700, P700*, and P700* then accumulates, although both the pmf and RISE are kept to the same or a slightly greater extent or as in Case (II). That is, the suppression of the apparent rate constant of the oxidation of P700* is larger than that of the oxidation activity of the reduced PQ in the Cyt *b*_6_/*f*-complex in response to the limitation of the electron sink. Electrons accumulate at the acceptor side of PSI, and the electrons flow to O_2_ to produce O_2_**^−^**, the production of ROS. The transition of the rate-determining step from the reduction of P700^+^ to the oxidation of P700* is a dangerous condition that induces oxidative damage to PSI.

### 4.5. Relationship between Photorespiration- and Flavodiiron Protein (FLV)-Dependent Electron Flows and Their Contribution to P700 Oxidation in PSI

Following the model of the P700 oxidation system, photosynthetic linear electron flow contributes to the induction of either the pmf or RISE, which suppresses the electron flow from the Cyt *b*_6_/*f*-complex to the oxidized P700 through PC. Afterwards, P700 is oxidized, as shown in Figure 5 and Figure 6. From cyanobacteria to gymnosperms, in the evolution of green lineage oxygenic photosynthetic organisms, flavodiiron protein (FLV) drives O_2_-dependent electron flow in the photosynthetic electron transport system, in addition to the photorespiration pathway [88,89,90,94,95,96]. FLV reduces O_2_ to H_2_O using NADPH as the electron donor [88]. Different from photorespiration, FLV-dependent electron flow does not consume ATP; i.e., it only induces the pmf. Therefore, FLV-dependent electron flow has a higher capacity to oxidize P700. In fact, in the cyanobacteria *Synechocystis* sp. PCC6803 and *Synechococcus* sp. PCC7002, the deletion of *flv* genes caused the photoinhibition of PSI, which was enhanced by the reduction of P700 in PSI [58]. Furthermore, the liverwort *Marchantia polymorpha*, which was deficient in flv genes, suffered from the photoinhibition of PSI due to the reduction of P700 [58]. Angiosperms, however, do not have FLV genes in their genomes [87,97]. Among land plants, the electron flux of photorespiration-dependent photosynthetic linear electron flow was compared to FLV-dependent electron flux at the CO_2_ compensation point, where photosynthetic activity was suppressed to zero. The maximum activities of both FLV-dependent and photorespiration-dependent photosynthetic linear electron flow were then evaluated among land plants [98].

**Figure 7:**
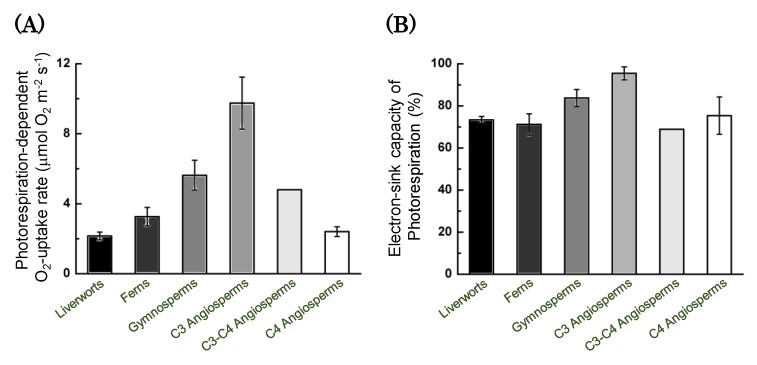
Evolution of tight coupling between the light and the dark reactions in photosynthesis. Upon spreading on the surface of the earth, oxygenic photosynthetic organisms inevitably metabolized 2-phosphoglycolate to regenerate 3-phosphoglycerate via the photorespiration pathway, because ribulose 1,5-bisphophate (RuBP) carboxylase/oxygenase (Rubisco) catalyzes both RuBP carboxylation and oxygenation under atmospheric conditions. Consequently, photorespiration inevitably accompanies photosynthetic CO_2_ fixation. In C3 angiosperms, both photosynthetic CO_2_ fixation and photorespiration have become the main electron sinks in the photosynthetic process. Hanawa et al. [98] found that photorespiration activity increased with the evolution of oxygenic photosynthetic organisms. Photorespiration activity is evaluated as a photorespiration-dependent O_2_-uptake at the CO_2_ compensation point, at which point its activity peaks [66]. (**A**) Photorespiration activity increases with the evolution of oxygenic photosynthetic organisms from liverworts to C3 angiosperms through ferns and gymnosperms. (**B**) The electron sink capacity of photorespiration reaches its maximum in C3 angiosperms. The activities of flavodiiron (FLV)-dependent photosynthetic linear electron flow and other alternative photosynthetic linear electron flow (AEF) decrease upon the evolution of oxygenic photosynthetic organisms from liverworts to C3 angiosperms through ferns and gymnosperms. On the other hand, C3-C4 and C4 plants show lower photorespiration activity, and other electron sinks function at the CO_2_ compensation point. Data are from Hanawa et al. [98].

Photorespiration activities at the CO_2_ compensation point increased with the evolution of photosynthetic organisms from liverworts to C3 angiosperms to ferns and gymnosperms (Figure 7A). C3-C4 and C4 angiosperms show lower activities of photorespiration, compared to C3 angiosperms. C3 angiosperms show the maximum activities of photorespiration [98]. In C3 angiosperms, photosynthetic linear electron flow was mainly driven by photorespiration, which was the main electron sink in photosynthesis, at the CO_2_ compensation point (Figure 7B). An electron sink other than photorespiration was found in liverworts, ferns, and angiosperms. FLV could be an electron sink at the CO_2_ compensation point. C3-C4 and C4 angiosperms also had an electron sink other than photorespiration and FLV-dependent photosynthetic linear electron flows, but its mechanism is unknown.

What do C3-angiosperms achieve by discarding FLV genes in their genomes? Different from photorespiration, FLV-dependent electron flow does not consume ATP; it produces it, as described above. FLV-dependent electron flow induces the pmf, which can be a self-regulating electron flow and suppress photosynthesis, because an enhanced pmf suppresses the activity of the Cyt *b*_6_/*f*-complex, as shown in Figure 5 and Figure 6. In liverworts, ferns, and gymnosperms, FLV-dependent photosynthetic linear electron flow functions immediately after the actinic light illumination starts, and its flux is suppressed at the steady state of photosynthesis [98]. This indicates that FLV-dependent photosynthetic linear electron flow is generally not essential to photosynthesis organisms, and that there is a molecular mechanism to reduce FLV-dependent photosynthetic linear electron flow under a steady state of photosynthesis. On the other hand, photorespiration drives both the light reaction and the dark reaction, and inevitably functions with photosynthesis. This situation implies that a tight coupling of the light reaction with the dark reaction driven by both photosynthesis and photorespiration can regulate both the pmf and RISE in response to their activities, because the dark reaction induces and dissipates the pmf and RISE simultaneously. This could be a sensitive trigger for the regulation of P700 oxidation. If FLV-dependent photosynthetic linear electron flow participates in the photosynthetic linear electron flow driven by both photosynthesis and photorespiration, the automatic regulation of photosynthetic linear electron flow will collapse. Therefore, C3 angiosperms do not require FLV-dependent photosynthetic linear electron flow, and discard FLV genes in their genomes. These conclusions explain why angiosperms can have a high activity of photosynthesis, if photosynthetic organisms having FLV genes in their genomes have lower photosynthetic activity.

## 5. Conclusions

Oxygenic photosynthetic organisms have a robust, common molecular mechanism to suppress the production of reactive oxygen species (ROS), the P700 oxidation system, because the ROS-dependent oxidative inactivation of PSI is lethal to these organisms. When cyanobacteria first appeared on the Earth about 2–3 billion years ago, they already had a P700 oxidation system [58]. Photosynthetic organisms regulate the activity of plastoquinol oxidation by the cytochrome (Cyt) *b*_6_/*f*-complex using two strategies: pmf formation and the reduction-induced suppression of electron flow (RISE), both of which are used to suppress the electron flux from the Cyt *b*_6_/*f*-complex to the reaction center chlorophyll P700 in PSI. The induction of both the pmf and RISE is sensitive to changes in the activities of both photosynthesis and photorespiration in land plants, which drive photosynthetic linear electron flow. The magnitudes of both the pmf and RISE are determined by the activity of photosynthetic linear electron flow. Upon exposure to environmental stress, e.g., low temperature, and/or drought, suppressed activities of both photosynthesis and photorespiration cause both the pmf and RISE to oxidize P700 in the manner described in the text for the suppression of ROS production in PSI.
